# Xylitol as a Hydrophilization Moiety for a Biocatalytically Synthesized Ibuprofen Prodrug

**DOI:** 10.3390/ijms23042026

**Published:** 2022-02-11

**Authors:** Federico Zappaterra, Chiara Tupini, Daniela Summa, Virginia Cristofori, Stefania Costa, Claudio Trapella, Ilaria Lampronti, Elena Tamburini

**Affiliations:** 1 Department of Environmental and Prevention Sciences, University of Ferrara, Corso Ercole I d’Este, 32-44121 Ferrara, FE, Italy; zppfrc@unife.it (F.Z.); daniela.summa@unife.it (D.S.); tme@unife.it (E.T.); 2 Department of Life Sciences and Biotechnology, University of Ferrara, Via Luigi Borsari, 46-44121 Ferrara, FE, Italy; tpnchr@unife.it (C.T.); ilaria.lampronti@unife.it (I.L.); 3 Department of Chemical, Pharmaceutical and Agricultural Sciences, University of Ferrara, Via Luigi Borsari, 46-44121 Ferrara, FE, Italy; virginia.cristofori@unife.it (V.C.); trpcld@unife.it (C.T.); 4 Laboratorio per le Tecnologie delle Terapie Avanzate (LTTA), Via Fossato di Mortara, 70-44121 Ferrara, FE, Italy

**Keywords:** ibuprofen, xylitol, CALB, prodrug, esterification, cystic fibrosis (CF), IB3-1

## Abstract

Biocatalyzed synthesis can be exploited to produce high-value products, such as prodrugs. The replacement of chemical approaches with biocatalytic processes is advantageous in terms of environmental prevention, embracing the principles of green chemistry. In this work, we propose the covalent attachment of xylitol to ibuprofen to produce an IBU-xylitol ester prodrug. Xylitol was chosen as a hydrophilizer for the final prodrug, enhancing the water solubility of ibuprofen. Ibuprofen is a nonsteroidal anti-inflammatory drug (NSAID) extensively used as an analgesic, anti-inflammatory, and antipyretic. Despite being the third-most-prescribed medicine in the world, the aqueous solubility of ibuprofen is just 21 mg/L. This poor water solubility greatly limits the bioavailability of ibuprofen. We aimed to functionalize ibuprofen with xylitol using the reusable immobilized N435 biocatalyst. Instead of a biphasic media, we proposed a monophasic reaction environment. The characterization of the IBU-xylitol ester was performed by ^1^H, ^13^C-NMR, DEPT, COSY, HMQC, HMBC, FTIR, and MS spectroscopy. Preliminary in vitro tests showed that this enzymatically synthesized prodrug of ibuprofen reduced the expression of the interleukin 8 genes in human bronchial epithelial cells (IB3-1) from cystic fibrosis (CF) patients.

## 1. Introduction

Sugar alcohols are polyols obtained by the reduction of sugars [1]. Polyols, such as glycerol, erythritol, xylitol, and sorbitol, are hydrogenated carbohydrates often used as sugar substitutes [2,3]. Xylitol (pentane-1,2,3,4,5-pentanol) naturally occurs in fruits and vegetables, such as plums, strawberries, cauliflower, and pumpkin [4]. Polyalcohols, such as xylitol, with their great water solubility, can be used as hydrophilization moieties to produce enhanced water-soluble prodrugs. Around 40% of new chemical entities (NCE) possessing pharmacological activities are poorly soluble, or even insoluble. Low water solubility represents a great obstacle in drug and formulation development [5]. For this reason, much attention is being paid to the development of protocols to produce highly water-soluble prodrugs. Chemically catalyzed esterification reactions between hydroxyl-carrying molecules and poorly water-soluble drugs were exploited for prodrug synthesis. A glycosylated ursodeoxycholic acid (UDCA) was tested with regard to its pharmacological effects in cholestatic rat models resulting in a higher capacity of reduction of serum biomarkers and cytokines compared to native UDCA [2,3]. Similar hydrophilization strategies can also be employed with anti-inflammatory, poorly bioavailable drugs, such as ibuprofen.

Ibuprofen, developed in the 1960s [6], is a widely used anti-inflammatory drug (NSAID) [7]. Since its synthesis, ibuprofen has been used for the therapy of arthritis and its symptoms, such as swelling, pain, and stiffness [8]. Ibuprofen, as well as ketoprofen and flurbiprofen, belong to the nonsteroidal drugs used for their analgesic, anti-inflammatory, and antipyretic properties [9]. It is typically used for mild/moderate pain caused by dysmenorrhea, headaches, dental pain, postoperative pain, or pain caused by osteoarthritis, rheumatoid arthritis, and ankylosing spondylitis [10,11]. Ibuprofen can be administered for the treatment of several disorders, considering its well-known mechanism of action. Ibuprofen causes the non-selective reversible inhibition of cyclo-oxygenase isozymes COX-1 and COX-2, which are responsible for the conversion of arachidonic acid into prostaglandins [12].

Although ibuprofen is the third-most-prescribed counter medicine in the world [13], the bioavailability of this drug is limited by its water solubility of just 21 mg/L [14]. Due to this aspect, ibuprofen ranks among the poorly water-soluble drugs, characterized by dissolution-limited oral bioavailability [15]. The administration of ibuprofen at high doses can cause undesirable adverse effects due to the low rate of dissolution of the currently available solid dosage forms, and the consequent poor bioavailability of ibuprofen [16]. The chronic use of ibuprofen may cause severe unwanted adverse effects, such as gastric mucosal damage, specifically stomach ulceration, bleeding, and perforation [17]. The carboxylic acid of ibuprofen limits its solubility in acidic media, such as that of the stomach [18]. The direct contact of the acidic carboxylic group of ibuprofen with the gastric mucosa causes damage by a combination of local irritation and the local inhibition of the cytoprotective action of prostaglandins [19,20]. To overcome the limitations of ibuprofen, prodrugs can be designed.

The synthesis of ibuprofen prodrugs with highly water-soluble molecules can lead to a double effect; improving the bioavailability of ibuprofen and masking the acidic group of ibuprofen to decrease the gastrointestinal toxicity due to the direct contact effect [21]. Therefore, an esterification reaction between the carboxylic acid of ibuprofen and alcohol could be a strategy to synthesize a prodrug enhanced in its water solubility [22]. Prodrugs are compounds designed to undergo biotransformation reactions before displaying their pharmacological properties [23].

Among the several uses of ibuprofen, its anti-inflammatory activity is exploited in cystic fibrosis (CF). Indeed, ibuprofen has been shown to be capable of significantly slowing the progression of lung disease in patients with CF [24]. Cystic fibrosis is a monogenic disorder, most common in Caucasians, estimated to affect one out of 2.500–4.000 newborns. This disease is caused by gene mutations of the Cystic Fibrosis Transmembrane conductance Regulator (CFTR), which encodes a chloride channel expressed at the apical membrane of the epithelial cells [25]. CF patients are characterized by a progressive pulmonary obstruction, caused by the lack of CFTR chloride channel activity, that leads to constant neutrophil-dominated endobronchial inflammation and overwhelming bacterial infection. Chronic progressive pulmonary disease and respiratory failure leading to both morbidity and mortality in more than 90% of patients remain [26].

Due to the severe and persistent bronchial inflammation, anti-inflammatory therapies are of particular interest for CF lung disease [27]. Ibuprofen increases the function of both the wild-type cystic fibrosis transmembrane conductance regulator and the phenylalanine 580 deletion (∆F508) in cultured human airway epithelial cells [28]. High-dose ibuprofen, administrated in an animal model of chronic infection, reduced inflammation without hindering bacterial clearance [29]. Ibuprofen rescues mutant cystic fibrosis transmembrane conductance regulator trafficking [30]. However, the treatment complexity is challenging. Therefore, due to the need for anti-inflammatory therapies in CF, the creation of new anti-inflammatory drugs or derivatives of existing drugs has gained interest [27].

The esterification reaction involving ibuprofen to produce its prodrug could be performed chemically by a Fischer esterification [31]. Alternatively, esterification reactions exploiting biotechnological approaches can represent green ways for the synthesis of bioactive derivatives of ibuprofen [32]. To embrace green chemistry, the processes must use eco-friendly, non-toxic, and reusable catalysts to produce valuable chemical compounds [33]. For this purpose, lipases can be used to perform a biocatalyzed esterification reaction. Lipases (triacylglycerol hydrolases, EC 3.1.1.3) are the most used biological tool for enzymatic esterification. Indeed, since the 1990s, lipases have allowed researchers and industries to synthesize the ester bond under mild reaction conditions, with no need for co-factors, and with stereo-specificities to a wide range of substrates [34]. An essential feature for the reaction is their synthetic activity in non-aqueous solvents. Lipases can biocatalyze esterification or transesterification reactions, and they are commercially available in free or immobilized forms [35].

**Scheme 1:**
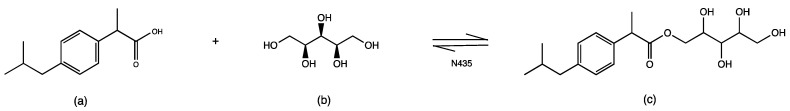
Esterification scheme of (**a**) ibuprofen racemate with (**b**) xylitol catalyzed by N435 (*Candida antarctica* lipase type B, immobilized) in monophasic media; (**c**) ibuprofen xylitol ester.

In this work, xylitol was chosen as a hydrophilizer to enhance the water solubility of ibuprofen. To this end, we aimed to covalently attach xylitol to ibuprofen, exploiting esterification reactions to synthesize a new derivative of ibuprofen (IBU-xylitol ester). Instead of chemical catalysts, we used lipases as biocatalysts. Between transesterification and direct esterification, we chose direct esterification catalyzed by commercially available immobilized *Candida antarctica* lipase type B (Novozym 435, N435—Scheme 1). The use of an immobilized biocatalyst allows its separation and re-use for several reaction cycles, promoting its choice as an adequate catalyst for the development of green processes. Xylitol has been previously used to synthesize prodrug xylitol butyrate derivatives as histone deacetylase inhibitors [36]. The reaction conditions, such as the lipase and compound concentrations, solvent amounts, temperatures, stirring speeds, and times are reported here. The structure of the new derivative of ibuprofen, xylitol ester of ibuprofen (IBU-xylitol), was studied with ^1^H, ^13^C-NMR, COSY, HSQC, HMBC, uHPLC-MS, and IR. Furthermore, its anti-inflammatory activity was tested with the bronchial epithelial IB3-1 cell line from CF, measuring the expression of the IL-8 gene by RT-PCR.

## 2. Results and Discussion

The approach used in this work aimed at the functionalization of the ibuprofen molecule, embracing the principles of green chemistry, using bioreactors designed to produce high-value products, and exploiting biocatalysts whose molecular mechanisms can produce prodrugs from a perspective of environmental prevention. Our strategy aimed to develop an effective esterification protocol to produce an ester between ibuprofen and xylitol using the lipase N435.

Xylitol, besides being freely soluble in water, could bring further molecular benefits to the final prodrug. This polyalcohol could not only characterize the hydrophobic portion of the final prodrug, but also could have a metabolic function. Indeed, it is reported that there are clinical trials that see the use of xylitol as a treatment for patients with cystic fibrosis [37,38].

### 2.1. Enzymatic Synthesis of IBU-Xylitol Ester

The literature has not reported cases of enzymatic esterification with characteristics, such as the protocol reported in the present work. Generally, the reactions that have been reported in the literature, which see the use of lipases, do not concern systems in which this biocatalyst must produce an ester bond between molecules with a partition coefficient (LogP) so different between the two esterification substrates (Table 1).

Indeed, as reported in Table 1, these molecules are opposite concerning their polarity. Ibuprofen is a strongly lipophilic molecule with a logP of 3.97, while xylitol is a strongly polar molecule and, consequently, very soluble in water. It is precisely the low polarity of ibuprofen that limits its water solubility and, therefore, its bioavailability.

In enzymatic esterification reactions, the lipase needs a reaction environment able to guarantee the mass transfer between the substrates and the catalyst [39]. Especially when the lipase is in its immobilized form, the proper solubilization of the substrates in the reaction media is essential. Typically, esterification reactions of molecules of opposite polarity are addressed using biphasic media reaction environments. In this case, an organic solvent solubilizes the lipophilic molecule, while water is used as a solvent for the hydrophilic molecule. However, this biphasic system requires fine reaction management and is intrinsically limited by the presence of water. Indeed, water, when present, can act as a nucleophile resolving the acyl–enzyme intermediate and leading to hydrolyses of the ester bond. Therefore, biphasic media esterification, when exploitable, is a hard-to-manage system implicitly limited by the poor mass transfer and the presence of water.

Literature reported a lipase-catalyzed esterification reaction in monophasic media. However, these reactions utilize mostly alcohols, even long-chain alcohols, which characterize the entire reaction environment taking part in the esterification reaction. Moreover, lipases are often used for racemic resolutions. In this context, methyl, ethyl, and propyl esters of ibuprofen were reported [40,41]. These esters, due to the aliphatic portion added to ibuprofen, enhance its lipophilicity instead of its aqueous solubility, making these synthetic protocols useless for our experimental purposes. Monophasic esterification reactions were used to enhance the solubility of ibuprofen. Ravelo et al. reported the solventless esterification of ibuprofen with glycerol [42]. This interesting protocol benefits from the viscous and liquid nature of this short polyalcohol. Nevertheless, the solubility advantage brought by the attachment of glycerol was limited by its just three hydroxyl groups (two in the final prodrug). Although this lipase-catalyzed esterification does not require any organic solvents, these systems are not workable for polyalcohols with a solid nature, such as xylitol. Xylitol, unlike glycerol, can bring four additional hydroxyl groups to the final prodrug, strongly enhancing its aqueous solubility.

To bypass the limitation of both biphasic systems and esterification systems involving glycerol or aliphatic alcohols (for example, methanol, ethanol, propyl alcohol) as media, we aimed to develop an enzymatic protocol able to produce effective esterification between ibuprofen and xylitol. For this purpose, we started with the challenging choice of a suitable organic solvent. The range of organic solvents studied is wide and can be consulted in the methods section in Table 2.

Literature reports that solvents with logP < 2 are less suitable for a biocatalytic purpose [43]. In particular, it has been reported the effects of these solvents on enzyme activity and substrate specificity [44]. The hydration state of the catalyst is to be considered. Organic solvents that are highly polar can affect the amount of water in the aqueous layer around the catalyst, impacting its stability and the resulting conversion yield of the process [45]. Starting from this assumption, we evaluated the solubility of xylitol in five organic solvents with a logP higher than 2 (Table 2). However, none of them could solubilize xylitol. Therefore, to guarantee the development of a monophasic reaction environment, we extended the tests to another six organic solvents (Table 2). Among them, just 2-methylbutan-2-ol allowed the solubilization of xylitol. Ibuprofen, due to its high lipophilicity, proved to be freely soluble in this solvent. For this reason, this tert-alcohol was chosen as a suitable organic solvent for further tests. Moreover, among the organic solvents, the use of a tert alcohol is preferable because of its low toxicity [46]. The high boiling point of this alcohol makes it the perfect choice for a flexible biocatalyst, such as N435. Indeed, CALB can express biocatalytic activity in a wide range of temperatures (37–80 °C), also beyond the boiling point of several organic solvents (e.g., pentane).

In a monophasic reaction system, such as the reported one, the choice of a suitable catalyst is crucial. Indeed, a monophasic media does not display any interface, being constituted of a single solvent. In this scenario, lipases subjected to interfacial activation, could not reach the catalytically active form. Therefore, we have chosen the *Candida antarctica* lipase type B (N435) as biocatalyst. In fact, the N435 enzyme can be used for monophasic esterification reactions because it does not require interfacial activation. N435, the immobilized form of CALB, has a tiny lid that just partially closes the active site of this lipase [47], making interfacial activation unnecessary. This enzyme is present on the market in both its free and immobilized forms [48]. The immobilized form of CALB can improve its stability and catalytic activity, even in organic solvents [49]. CALB’s immobilized form is among the most stable commercialized enzymes, facilitating its industrial applications [50].

In this work, ibuprofen was used in the form of a racemate. Literature reports how CALB can preferentially esterify the R-enantiomer of ibuprofen [47,51]. So, in this case this aspect was not investigated. Furthermore, it has been previously reported that the hydroxyl groups of polyalcohols that take part in ester bond formation are the primary ones [52]. This was further evidenced by the NMR spectra of IBU-xylitol (Supplementary Materials). Patti et al. reviewed biocatalytic reactions applied to the desymmetrization of meso-compounds or symmetric prochiral molecules [53]. The reaction could produce a mixture of four possible diastereoisomers. In this study, the diastereomers were not chromatographically separable. Furthermore, no diesters or other enzymatic esterification products were observed. 1,3-diester would be more lipophilic than the monoesterified ibuprofen and would be held back by the c18 column, producing a peak at retention times higher than that of the monoglyceride. Under the reaction conditions reported in this manuscript, no diester as a by-product was found.

**Figure 1:**
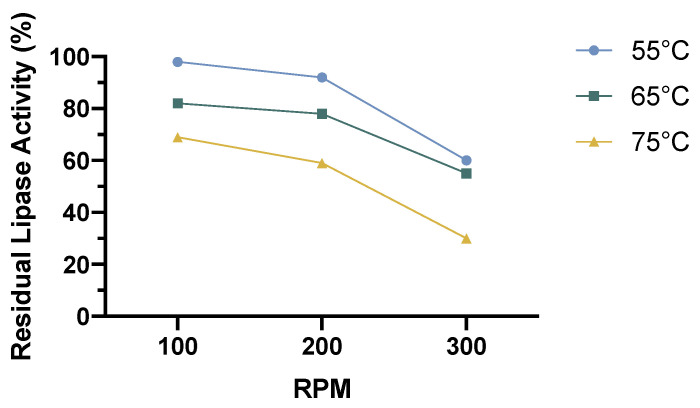
Effect of three different stirring speeds and temperatures on the residual lipase activity of N435 in 2-methylbutan-2-ol. Incubation time of 24 h.

Although lipases are reported as enzymes also active in environments where some amount of organic solvent is present, the effects of the solvent used need to be studied. Indeed, in an organic solvent monophasic environment, the agitation of the media and its temperature can produce instability in the biocatalyst. To assay the effect of these variables, the residual lipase activity was tested after 24 h of incubation time at different stirring speeds and temperatures. The results are shown in Figure 1.

Figure 1 reports the residual activity of N435 in the organic solvent chosen after 24 h of incubation time. The results in Figure 1 show that the temperature and stirring speed need to be managed to avoid enzymatic instability phenomena. In fact, with each increase in temperature, a decrease in the residual catalytic activity is observed. In addition, at equal temperatures, the increase in angular agitation also influenced the stability of the enzyme. At high temperatures, a greater decrease in the residual percentage activity was observed. From the results, although the temperature had a net impact on the enzymatic stability, the stirring of 300 RPM strongly impacted the stability of the biocatalyst. According to what was reported in Figure 1, we chose 200 RMP at 55 °C for further experiments.

**Figure 2:**
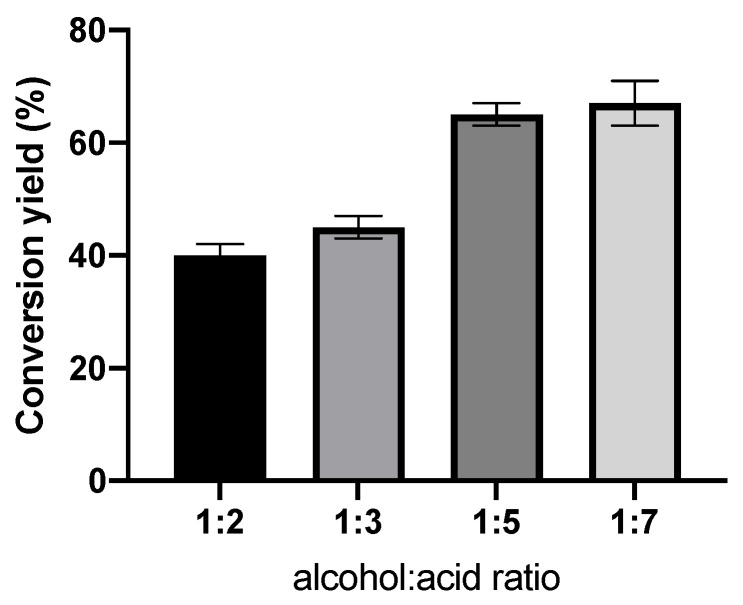
Effect of the alcohol/acid molar ratio on the conversion yield of IBU-xylitol ester catalyzed by N435. N435 concentration: 2 g L^−1^; 24 h reaction time.

As for the polyalcohol, the maximum solubility of xylitol in 2-methyl-butan-2-ol was assessed, reaching 10 g L^−1^. To generate a stoichiometric disequilibrium between substrates, which could enhance the synthesis of the product, we tested four different molar ratios. Since the hydroxyl groups can affect the stability of enzymes [54,55], ibuprofen was used in excess to shift the equilibrium of the reaction. The conversion yield was calculated by HPLC. The results are shown in Figure 2.

According to the results shown in Figure 2, in this highly concentrated environment, the stoichiometric disequilibrium of ibuprofen on xylitol enhanced the reaction of esterification until the ratio of 1:5. Beyond that, no significant improvement in the conversion yield was observed. Therefore, we chose a ratio of 1:5 for further experiments.

**Figure 3:**
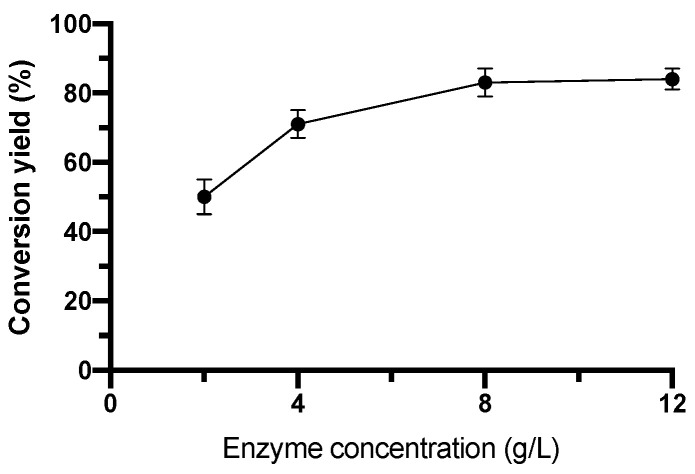
Effect of N435 concentration on the conversion yield of the IBU-xylitol ester.

To increase the conversion yield, the effect of the enzyme concentration was studied. Four concentrations between 2 and 12 g L^−1^ were tested. The results are shown in Figure 3.

Figure 3 shows that there was a correlation between the enzyme concentration and the percentage conversion yield of IBU-xylitol ester. The hyperbolic trend resulting from the increase of the N435 concentration is a typical curve of the reaction catalyzed by lipases [52,56]. The trend was due to the increase in the number of active sites accessible for the synthesis of the ester bond when the lipase concentration grew. The higher the concentration of enzyme, the greater the bond formation between the substrates, and the reaction rate increased. However, 12 g L^−1^ did not greatly enhance the conversion yield of the IBU-xylitol ester. Therefore, the most convenient concentration to use was 8 g L^−1^, reaching an 80% conversion yield. The immobilized nature of the enzyme chosen allowed its separation by the media after the reactions. The re-use of the catalyst performed well. Indeed, N435 maintained a conversion yield of 68 ± 3% after five reaction cycles.

The increase in polarity caused by the covalent attachment of xylitol to ibuprofen was evidenced by the chromatographic systems used during the study of the enzymatic esterification reaction. The thin-layer chromatographies (TLC), used as a reaction check, showed that the retention factor of IBU-xylitol ester was significantly lower than that of native ibuprofen, passing from Rf 0.83 for the acid to Rf 0.38 for the ester. On the C18 column of HPLC, the elution time of ibuprofen was 7.5 min, compared to 4.9 min for the IBU-xylitol ester. The prodrug showed a logP of 1.9 ± 0.08 and an aqueous solubility 12× better than that of the native ibuprofen.

### 2.2. IBU-Xylitol Ester: NMR and MS Spectroscopy Characterization

N435 has been previously shown as a biocatalyst that preferentially synthesizes the ester bond on the primary hydroxyls of polyalcohol, e.g., glycerol or sorbitol. Other lipases, such as the porcine pancreatic lipase—PPL, have shown the same esterification behavior. PPL was reported as a biocatalyst for the regioselective acylation of the primary hydroxyl group of monosaccharides [57]. Furthermore, polyol glycerol has been widely reported to produce polyesters with hydroxyl pendant groups. N435 allowed catalyzing the direct polymerization of sorbitol and adipic acid at the monosubstituted carbon position [58]. However, since the lipase-catalyzed esterification of ibuprofen with xylitol has not been reported previously, we studied the ester with ^1^H-, ^13^C-NMR, COSY, DEPT, HMQC, and HMBC (Supplementary Materials, Figures S2–S7). The study of the NMR allowed us to attribute the resonances of the IBU-xylitol ester groups (Supplementary Materials, Figure S1). The ^1^H NMR spectra of the synthesized compound showed chemical shifts, which were characteristic of the anticipated structure of the compound. The NMR spectrometry results confirmed that the esterification reaction took place between the carboxylic acid group of ibuprofen and the primary hydroxyl group of xylitol. Indeed, the two-dimensional homo- and heterocorrelated spectra allowed us to study the region of IBU-xylitol of the ester bond. The spectra confirmed that the hydroxyl, which was preferentially attacked by the ester bond, produced by the lipase was the primary one (Supplementary Materials).

**Figure 4:**
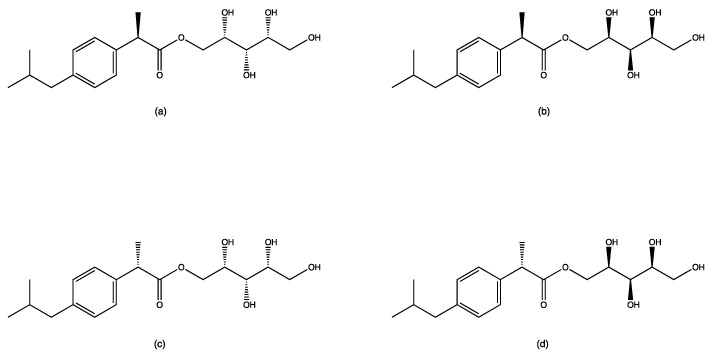
Stereochemistry of the four possible reaction products. Based on the chiral centers in the products, the mixture could present enantiomeric pairs (**a**)/(**d**), (**c**)/(**b**) and diastereomeric pairs (**a**)/(**b**), (**a**)/(**c**), (**d**)/(**b**), (**d**)/(**c**).

Since the experimental design presented in this work envisaged the use of ibuprofen in the racemate form, and that the attack on primary hydroxyls was equivalent from the point of view of lipase, the reaction products may have been a mixture of stereoisomers (Figure 4).

**Figure 5:**
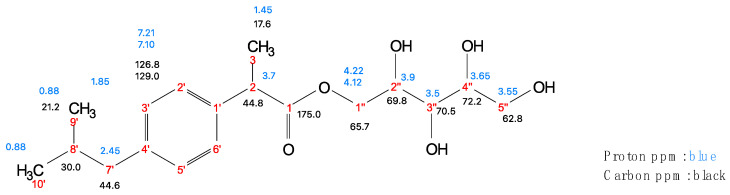
^1^H- and ^13^C-NMR attribution of the IBU-xylitol ester.

In this work, the stereochemistry of the reaction products was not investigated. Despite this, the NMR study allowed us to completely attribute the hydrogen and carbon chemical shifts. Figure 5 reports the unique chemical shifts of the proton and carbon NMR of the unique reaction product, regardless of the stereochemistry of the prodrug.

**Figure 6:**
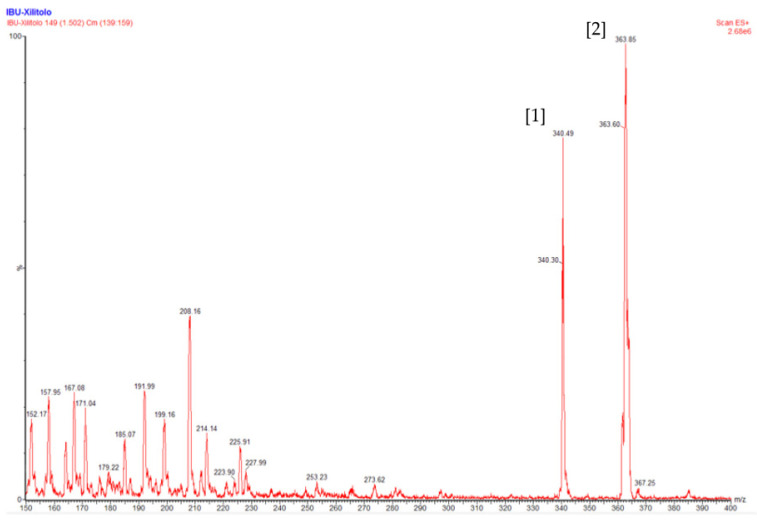
MS analysis of the esterification of ibuprofen with xylitol catalyzed by N435. ESI+/MS spectrum of the IBU-xylitol ester.

After the spectral analysis by NMR, we proceeded to study the mass of IBU-xylitol ester. The results are shown in Figure 6.

As reported in Figure 4, the mass spectra of the synthesized compounds confirmed the molecular weight of the compound. The IBU-xylitol ester had a predicted *m*/*z* of 340. The results shown in Figure 4, derived from the positive electrospray ionization (ESI+), outline the *m*/*z* of the IBU-xylitol ester (peak No. 1), as well as its adduct with sodium [M + Na]+; *m*/*z* 363 (peak No. 2), validating the presence of the enzymatically synthesized prodrug.

### 2.3. Preliminary In Vitro Tests of IBU-Xylitol Ester

In our experimental design, the choice of using polyalcohol xylitol as a hydrophilizing agent was determined by its high solubility in water, its well-known safety profile, and its metabolism once the parental drug has been regenerated [59]. Esterification is a process that can be used not only to produce a prodrug with increased solubility in water, but also to magnify the biological capabilities of the active ingredient of interest [60,61]. To this end, the rational design of a prodrug between ibuprofen and xylitol could be aimed at the synthesis of a novel ibuprofen derivative potentially interesting for the treatment of CF. Indeed, both ibuprofen and xylitol were previously tested in CF [37,38].

**Figure 7:**
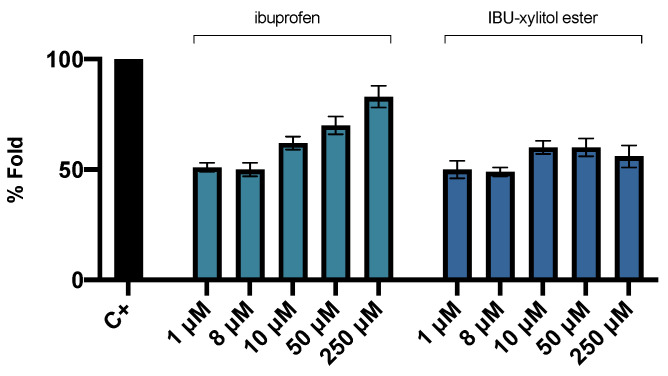
Fold change of the TNF-α induced IL-8 mRNA expression in IB3-1 cell line (C+) treated with Ibuprofen and IBU-xylitol ester at different concentrations (1–250 μM) and the relative anti-inflammatory effects, after 24 h incubation.

In this study, we utilized the IB3–1 bronchial cell line, prone to inflammatory states, expressing high levels of cytokines and chemokines. This cell line is often used for mimicking the inflammatory state frequently observed in the lungs of patients with CF [62]. To study if the prodrug IBU-xylitol ester could maintain the anti-inflammatory effect of ibuprofen, or even enhance it, we tested different concentrations of this derivative in CF bronchial epithelial IB3-1 cells treated with tumor necrosis factor-α (TNF-α) to gain an inflammatory state. The RT-PCR data analysis allowed us to assay the expression level of IL-8 in IB3-1 cells. The results are shown in Figure 7.

As shown in Figure 5, the prodrug IBU-xylitol ester proved to perform the anti-inflammatory effect in the IB3-1 cell line. This outcome confirmed that the covalent attachment of xylitol to ibuprofen did not inhibit the activity of the NSAID. At lower concentrations, ibuprofen and its ester showed the same anti-inflammatory capacity; when the concentration increased, the IBU-xylitol ester appeared to be more effective. The toxicity of the IBU-xylitol ester was less than that of the acid form of ibuprofen, with an IC_50_ of 550 μM for the IBU-xylitol ester and 500 μM for ibuprofen. These preliminary investigations are to encourage the development of the biocatalyzed esterification of NSAIDs as a potential strategy to enhance the water solubility of the active ingredients. The assay of the pro-apoptotic effects of the derivative of ibuprofen showed that the IBU-xylitol ester did not induce apoptosis when compared to the untreated cells (negative control, C-) (Supplementary Materials; Figure S9).

Often in CF, the chronic inflammation state is also dependent on the *P. aeruginosa* lung infection. The use of drugs able to mitigate the *Pseudomonas* effects, controlling its growth, could be interesting as a treatment for this disease. Therefore, we tested the antibacterial activity of IBU-xylitol ester against *P. aeruginosa*. However, the tests reported no inhibition of bacterial growth.

## 3. Materials and Methods

### 3.1. Materials

Novozym^®^ 435 (immobilized lipase B from *C. antarctica*) was kindly gifted by Novozymes A/S (Frederiksberg, Denmark). Ibuprofen sodium salt, racemic (≥98% pure), xylitol (≥98% pure), silica gel (60 Å, 70–230 mesh, 63–200 µm), and methanol-d4 degree 99.8% were purchased from Sigma–Aldrich (Buchs, Swiss). All other solvents, other than the methanol for HPLC, were of ACS grade. The heated magnetic stirrer was an “AREX Digital PRO,” from Velp Scientifica SRL (Milano, Italy). The nuclear magnetic resonance (NMR) spectra were recorded with a 400 MHz Varian Gemini spectrometer (Varian, Palo Alto, CA, USA). The IR spectra were recorded with a Perkin Elmer FTIR Spectrum 100 infrared spectrometer (Milano, Italy) equipped with ATR using a ZnSe Diamond.

### 3.2. Thin-Layer Chromatography (TLC)

As the method of reaction monitoring, TLC analyses were conducted to check the reaction process. In total, 100 µL of the reaction mixtures was diluted 1/10 in MeOH and analyzed on TLC plates (TLC Silica gel 60, 5 × 10 cm, Merck, Berlin, Germany). Chromatography was performed with a mobile phase containing ethyl acetate/hexane/acetic acid/MeCN 50:25:8:17 (*v*/*v*/*v*/*v*). After complete elution, the thin layer was dried and the separation results were acquired via UV spectroscopy (λ = 254 nm) followed by chemical derivatization with the phosphomolybdic acid solution. The xylitol ester of ibuprofen resulted in a retention factor (Rf) of 0.38.

### 3.3. Biocatalytic Synthesis of IBU-Xylitol Ester

For the synthesis of IBU–xylitol, several organic solvents were tested (Table 2).

The reactions were carried out in screw-capped 20 mL vials immersed in a glycerin bath placed on a magnetic stirrer equipped with a temperature probe capable of self-regulating the temperature of the heating plate to maintain the temperature over time. The magnet used as a stirrer for the media in the microreactor was a double-sided crosshead magnetic stirrer of 1.5 cm in diameter. After the enzymatic stability assessment, the effect of the esterification parameter was tested. Ibuprofen (338 mg) and xylitol (50 mg) were added to 5 mL of 2M2B with 40 mg of N435. The reaction was kept at 55 °C and 200 RPM for 24 h. The biocatalyst was washed 3 times with tetrahydrofuran, dried, and reused in a new reaction cycle.

### 3.4. Analytical HPLC Analysis

To determine the conversion yields of all the optimization tests, this was calculated by JASCO HPLC modular system equipped with a reverse-phase column (Synergi 4 µm Hydro-RP 80 Å−250 × 4.6 mm), a refractive index (model RI-4030), and a UV–Vis detector (model UV-4070)—30 °C, mobile phase 73:17 MeOH/H_2_O (+0.1% H_2_SO_4_) at 0.8 mL/min. The conversion yield was calculated using the following equation:

$$X=\frac{A_{ester}}{A_{xylitol}-A_{ester}}$$

where $A_{ester}$ is the area of the IBU-xylitol ester and $A_{xylitol}$ is the area of xylitol [63]. Negative controls of the reaction were prepared without the use of lipase and all of the experiments were conducted in triplicate.

### 3.5. Analytical uHPLC-MS Method

uHPLC–MS analysis was conducted using a Waters Acquity uHPLC with ZQ 2000 ESI mass spectrometry (Waters, Milford, MA, USA) and Mass Link software (Waters, Milford, MA, USA). The 2.6 µm Kinetex 50 mm × 4.6 mm C18 column was selected to perform the analysis. The mobile phases used were water with 0.1% formic acid as solvent A and acetonitrile with 0.1% formic acid as solvent B. The liquid chromatography ran in a gradient condition from 100% H_2_O at t0 to 100% acetonitrile at t5 (5 min) under a flow rate of 0.3 mL/min. The column operated at a stationary temperature of 40 °C. The temperature, nebulizer pressure, and flow rate of the drying gas (N2) were 230 °C, 35 psi, and 10 L/min, respectively. The further operation parameters were 1200 V for the nozzle voltage and 2500 V for the capillary voltage. Mass spectra were tracked in a mass–to–charge (*m*/*z*) ratio range of 150–500 in the positive ion-detection mode.

### 3.6. Purification and Spectroscopic Characterization of IBU-Xylitol Ester

The reaction products were separated by glass column chromatography. The column was prepared with silica gel using a mixture of ethyl acetate/hexane/acetic acid/MeCN 50:25:8:17 (*v*/*v*/*v*/*v*) as the elution solvent. The IBU-xylitol ester was isolated. The fractions containing the product were combined, the solvent was evaporated using a rotary evaporator, and the residue was analyzed by ^1^H and ^13^C NMR (400-MHz Varian Gemini spectrometer; Varian, Palo Alto, CA, USA). The NMR sample was prepared by dissolving 15 mg of IBU-xylitol in deuterated methanol (1 mL). The ^1^H- and ^13^C-NMR spectra showed that the compound’s structure was the expected one. The NMR spectra (Supplementary Materials) showed the following peaks: ^1^H-NMR (400 MHz, CD_3_OD) δ 7.21 (d, J = 8.2 Hz, 2H, H-3′, 5′), 7.10 (d, J = 8.0 Hz, 2H, H-6′, 2′), 4.22 (m, 1H, H-1″), 4.12 (m, 1H, H-1″), 3.93–3.75 (2H, H-2, 2″), 3.75–3.46 (m, 4H, H-3″, 4″, 5″), 2.45 (d, J = 7.2 Hz, 2H, H2-7′), 1.84 (hept, J = 6.6 Hz, 1H, H-8′), 1.46 (d, J = 7.2 Hz, 3H, H3-3), 0.89 (d, J = 6.6 Hz, 6H, H3-9′, H3-10′). ^13^C-NMR (101 MHz, CD_3_OD) δ 175.0, 140.3, 138.0, 128.9 (2C), 126.9 (2C), 72.2, 70.5, 69.9, 65.7, 65.4, 62.8, 44.8, 44.6, 30.0, 21.3, and 17.6.

IR (Figure S8): 3365.27, 2953.67, 1722.22, 1560, 1512.10, 1456.06, 1382.97, 1334.47, 1201.13, 1165.92, 1129.58, 1067.44, 1021.57, and 847.7.

### 3.7. Cell and Bacterial Line: Culture Condition

The immortalized IB3-1 cell line (LGC Promochem Europe) was used. These cells were derived from fibrocystic patients carrying the ΔF508/W1282X mutated genotype. The cells were grown in the basal medium LHC-8 (Biofluids, Rockville, MO, USA) supplemented with 5% FBS. The IB3-1 cells, after seeding in 24 well plates, were treated with ibuprofen and the IBU-xylitol ester (vehicle 75% EtOH). Five hours later, we stimulated the cells with TNF-α 100 ng/mL and incubated them for a further 24 h. After that, the supernatants were collected, and the total RNA was extracted. *P. aeruginosa*, the PAO1 laboratory strain, was kindly provided by A. Prince (Columbia University, New York). The bacteria were grown in trypticase soy broth (TSB) or agar (TSA) (Difco, Detroit, MI, USA).

### 3.8. Cytotoxicity Determination-IC50

IB3-1 cells were seeded at a density of 50,000 cells/mL in 24-well plates in LHC-8 medium in the presence of 5% FBS. At the exponential growth phase, the cells were treated with the compounds. After 24 h, the cells were washed with PBS 1X, detached with trypsin/EDTA, and resuspended in LHC-8 medium. Finally, the cells were counted with a Coulter Counter; model Z Series; Beckman, Brea, CA, USA.

### 3.9. Anti-Bacterial Assay

The anti-microbial activity of the IBU-xylitol ester and ibuprofen was determined by following the procedure of the Broth Dilution Method [64]. *P. aeruginosa* (PAO1 strain) was cultured on agar plates of TSA overnight at 37 °C. PAO-1 was diluted in 7 mL of TBS, adjusting the starting concentration to 0.5 MCFU. To 2 mL TBS in a tube, 1.4 µL of PA0-1 suspension was added. One tube contained only 2 mL of TBS as a negative control. Tubes with 2 mL of TBS and concentrations of derivatives were prepared. The samples were read on a densitometer.

### 3.10. RNA Extraction and IL-8 Gene Expression Analysis (RT-qPCR)

The isolation of the total RNA was carried out from confluent cells using TRIzol Reagent (Invitrogen, Carlsbad, CA, USA) following the manufacturer’s protocol. The quantification of the isolated RNA was performed by a UV-ray spectrophotometer (model SmartSpec Plus; BioRad, Hercules, CA, USA). One microgram of RNA was used for the cDNA synthesis using the ImProm-II Reverse Transcription System cDNA synthesis kit (Promega, Madison, WI, USA). The cDNA was PCR amplified with a CFX96 Touch Real-Time PCR Detection System (Thermal Cycler model C1000 Touch; BioRad, Hercules, CA, USA) using SYBR^®^ Green Supermix 1x (Abs 520 nm BioRad; Hercules, CA, USA). The PCR was performed with the first denaturation step at 95 °C for 30 s and then carried out for 40 cycles at 95 °C for 5 s and 60 °C for 30 s. The relative levels of each sample were calculated by the 2^−ΔΔCT^ method [65] and normalized with GAPDH housekeeping genes.

The gene-specific primers and housekeeping are in Table 3.

### 3.11. Apoptosis Analysis

A Muse^®^ Cell Analyzer (Merck Millipore, Burlington, MA, USA) was used to perform the Annexin V assay on IB3-1 cells according to the manufacturer’s protocol. Cells were exposed to the IBU-xylitol ester and ibuprofen of 500 µM for 24 h. This procedure was based on the detection of PS (PhosphatidylSerine) exposed on the external membrane of apoptotic cells by Annexin V and on the revealing of the 7AAD (7-Amino Actinomycin D) DNA-stain. Muse Annexin V & Dead Cell reagent was used to dilute the cells (1:1). After 20 min of incubation between the cell samples and kit at room temperature in the dark, the samples were analyzed. The data were acquired and recorded utilizing the Annexin V and Dead Cell Software Module (Millipore, Billerica, MA, USA).

### 3.12. Statistical Analysis

All the results were expressed as the means ± SEM. The treatments, sampling time, and their interaction were tested by parametric (one-way ANOVA) and non-parametric (Mann-Whitney and Kruskal-Wallis tests) tests. Bonferroni’s Multiple Comparison Test was applied as a post hoc test. *p* values < 0.05 were considered statistically significant. The data were analyzed using the software GraphPad Prism 4.0 (GraphPad Software, Inc., La Jolla, CA, USA).

## 4. Conclusions

This study reports the optimized enzymatic esterification of ibuprofen with xylitol. Lipase B from *Candida antarctica* (N435) is a suitable enzyme for the synthesis of the IBU-xylitol ester in a monophasic reaction environment. To the best of our knowledge, this is the first report of the synthesis of the IBU-xylitol ester prodrug and the first protocol that presents productive enzymatic esterification of ibuprofen with a highly polar polyalcohol, such as xylitol, in a monophasic batch environment. The optimization of the reaction parameters allowed the system to remain stable and to perfectly recover and reuse the biocatalyst from the media. This prodrug was characterized by ^1^H, ^13^C-NMR, DEPT, COZY, HMQC, HMBC, FTIR, and MS spectroscopy. RT-PCR analyses were performed on the IB3-1 cell line and showed that the enzymatic esterification did not limit the anti-inflammatory capacity of the parental drug. The IBU-xylitol ester could be an interesting derivative of ibuprofen for the treatment of cystic fibrosis.

## Figures and Tables

**Table 1:** Chemical characteristics of the substrates of esterification.

Substrate	Chemical Formula	Molecular Weight	Melting Point (°C)	LogP
Ibuprofen	C_13_H_18_O_2_	206.29	76	3.97
Xylitol	C_5_H_12_O_5_	152.15	93.5	−2.56

**Table 2:** Proprieties of the organic solvents tested.

Solvent	LogP	Boiling Point (°C)
Acetonitrile	−0.34	81.6
Benzene	2.13	80
Cyclohexane	3.44	80.7
Dimethyl carbonate	0.23	90.5
Ethyl acetate	0.73	77.1
Hexane	3.9	68.7
Isoamyl alcohol	1.16	131.1
Petroleum ether	4.66	98.5
2-methylbutan-2-ol	0.89	102.4
Tetrahydrofuran	0.46	65
Toluene	2.73	110.6

**Table 3:** PCR primer sequences.

Gene	Primer Sequence	Tm °C	Product Length (bp)
*IL-8*	F: 5′-gtgcagttttgccaaggagt-3′	56.3	20
	R: 5′-ttatgaattctcagccctcttcaaaaact-3′	58.6	29
*GAPDH*	F: 5′-aaggtcggagtcaacggattt-3′	56.4	21
	R: 5′-actgtggtcatgagtccttcc-3′	56.5	21

## Supplementary Materials

The following are available online at https://www.mdpi.com/article/10.3390/ijms23042026/s1.
