# Supplementary material for: Xylitol as a Hydrophilization Moiety for a Biocatalytically Synthesized Ibuprofen Prodrug
Source: Int J Mol Sci. 2022 Feb 11;23(4):2026. doi: 10.3390/ijms23042026 (PMC8880498; doi:10.3390/ijms23042026)
Supplement: Supplementary file 1 [file ijms-23-02026-s001.zip › ijms-1546390-supplementary.pdf]

International Journal of
Molecular Sciences

MDPI

Supplementary Materials

Article

Xylitol as a Hydrophilization Moiety for a Biocatalytically Synthesized Ibuprofen Prodrug

Federico Zappaterra, Chiara Tupini, Daniela Summa, Virginia Cristofori, Stefania Costa, Claudio Trapella, Ilaria Lampronti and Elena Tamburini

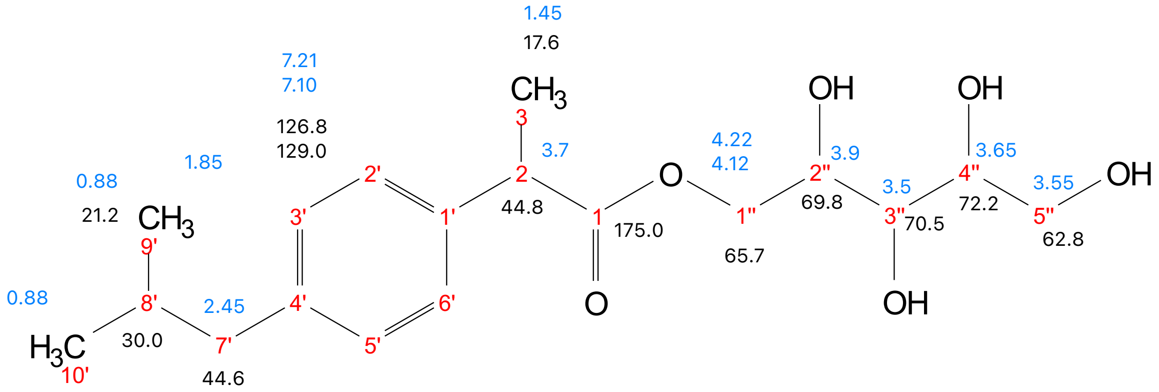

Proton ppm: blue
Carbon ppm: black

Figure S1: ^{1}H- and ^{13}C-NMR attribution of IBU-xylitol ester.

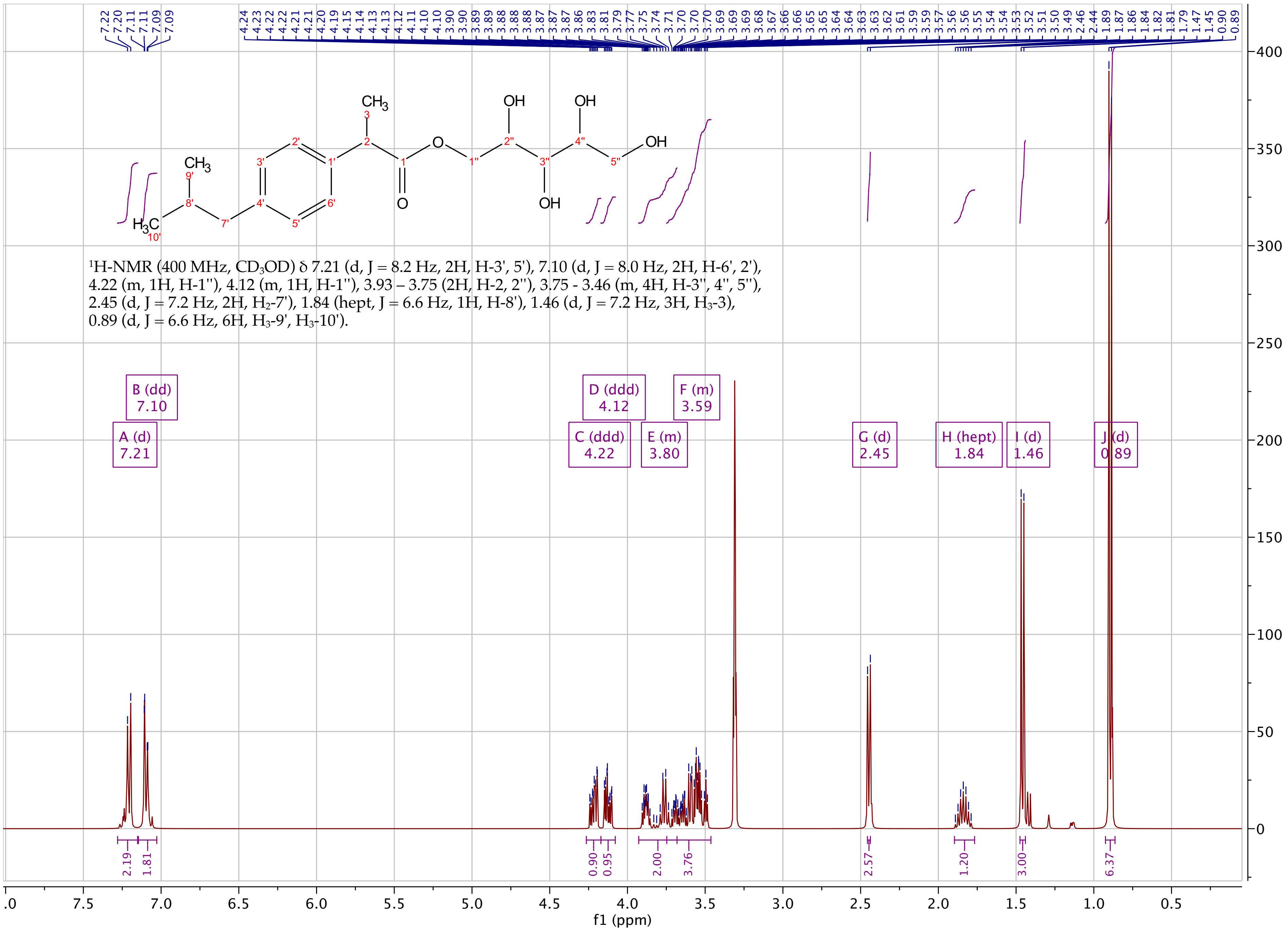

Figure S2: 1H-NMR spectra of IBU-xylitol ester; RT, Methanol-d4, 400 MHz.

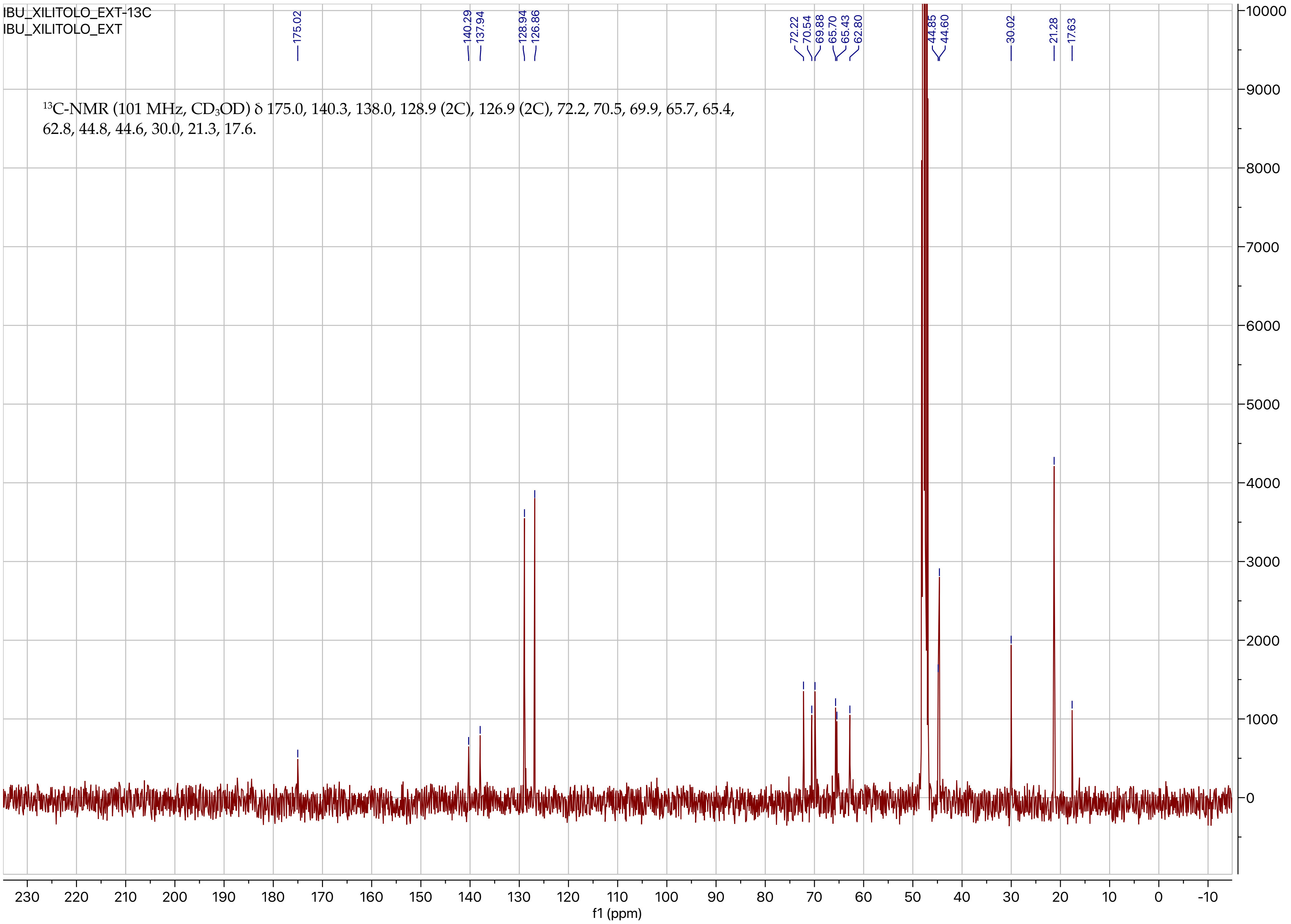

Figure S3: 13C-NMR of IBU-xylitol ester; RT, Methanol-d4, 101 MHz.

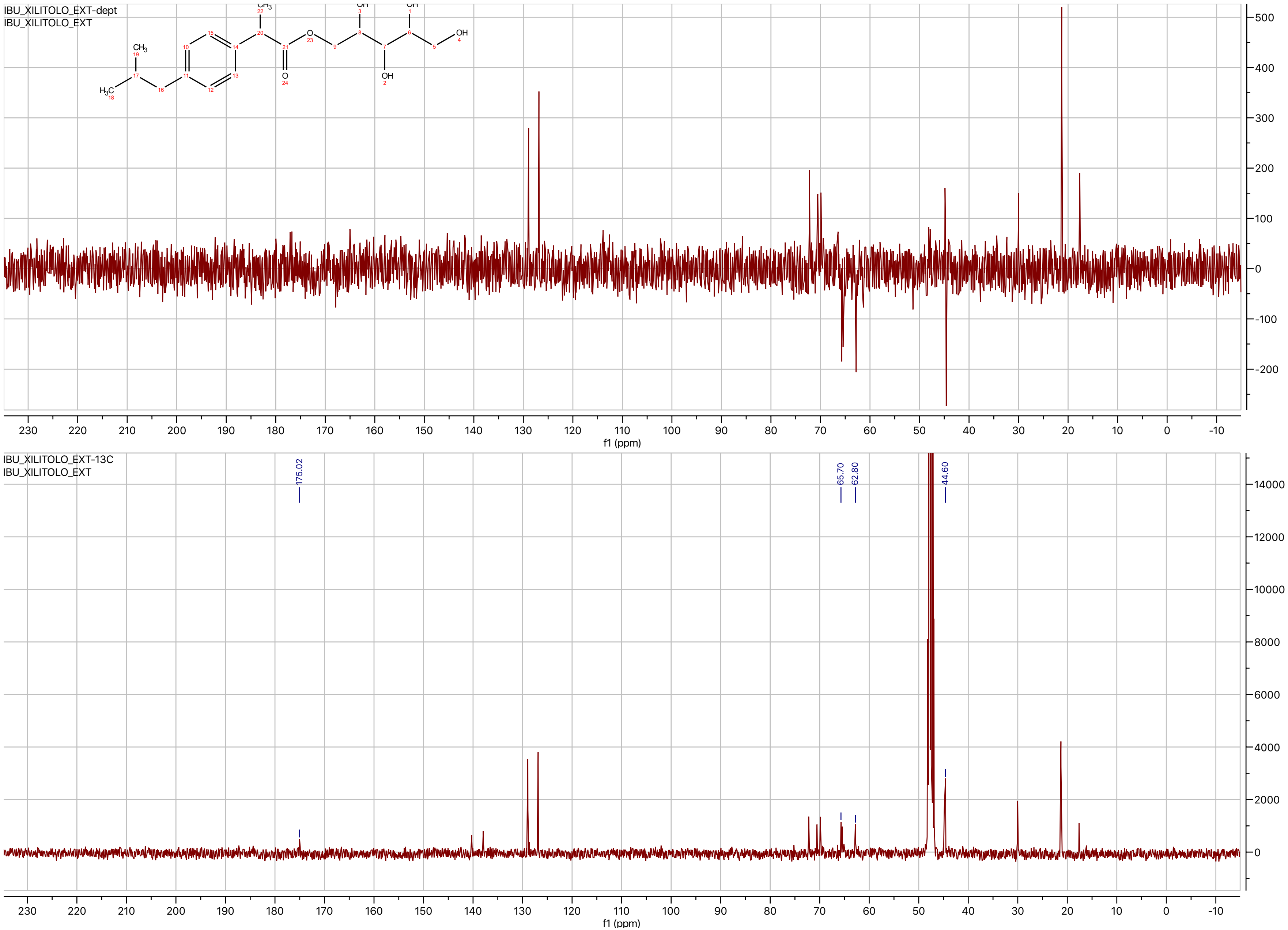

Figure S4: DEPT IBU-xylitol ester; RT, Methanol-d4, 101 MHz.

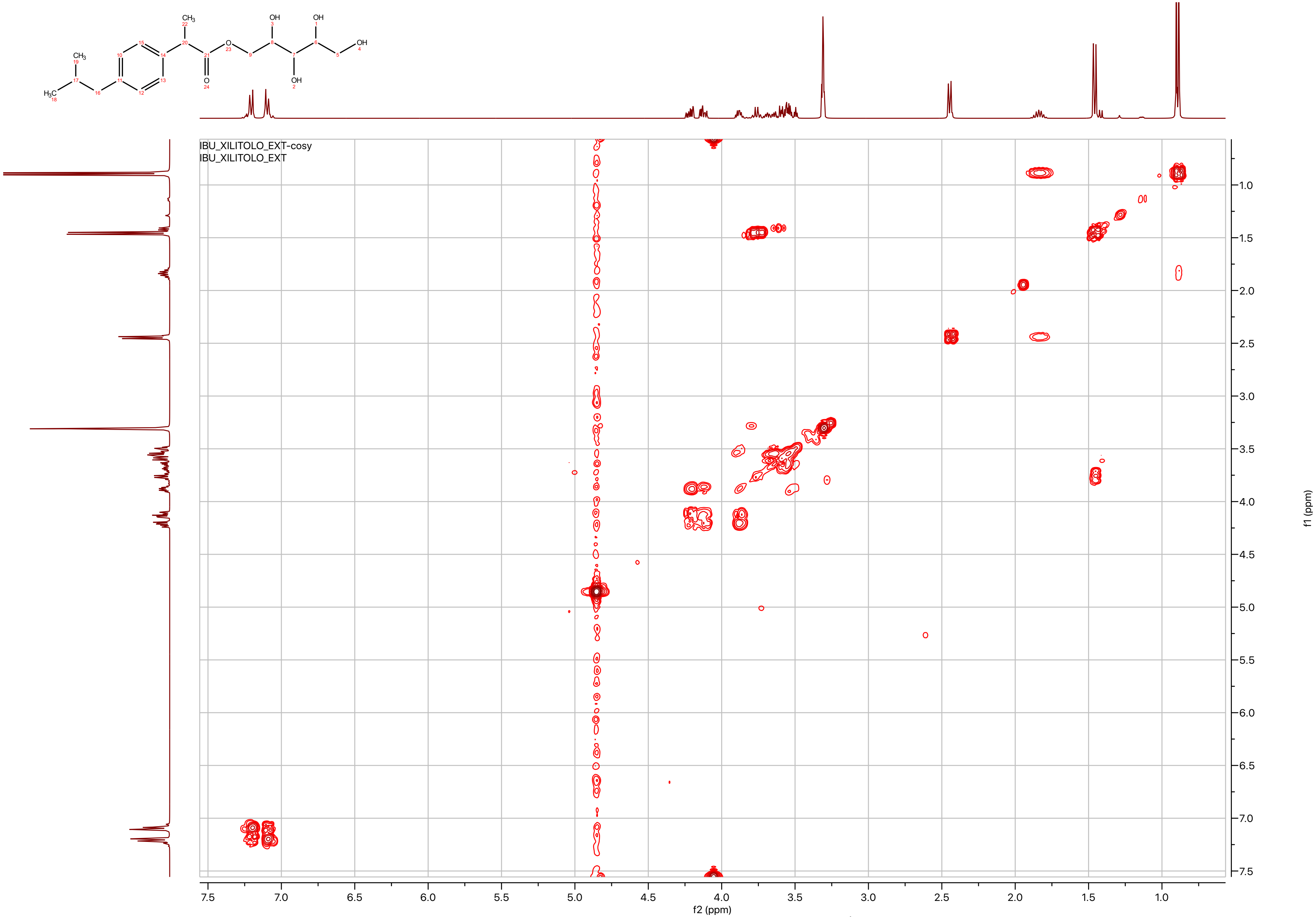

Figure S5: COSY IBU-xylitol ester; RT, Methanol-d4, 101 MHz

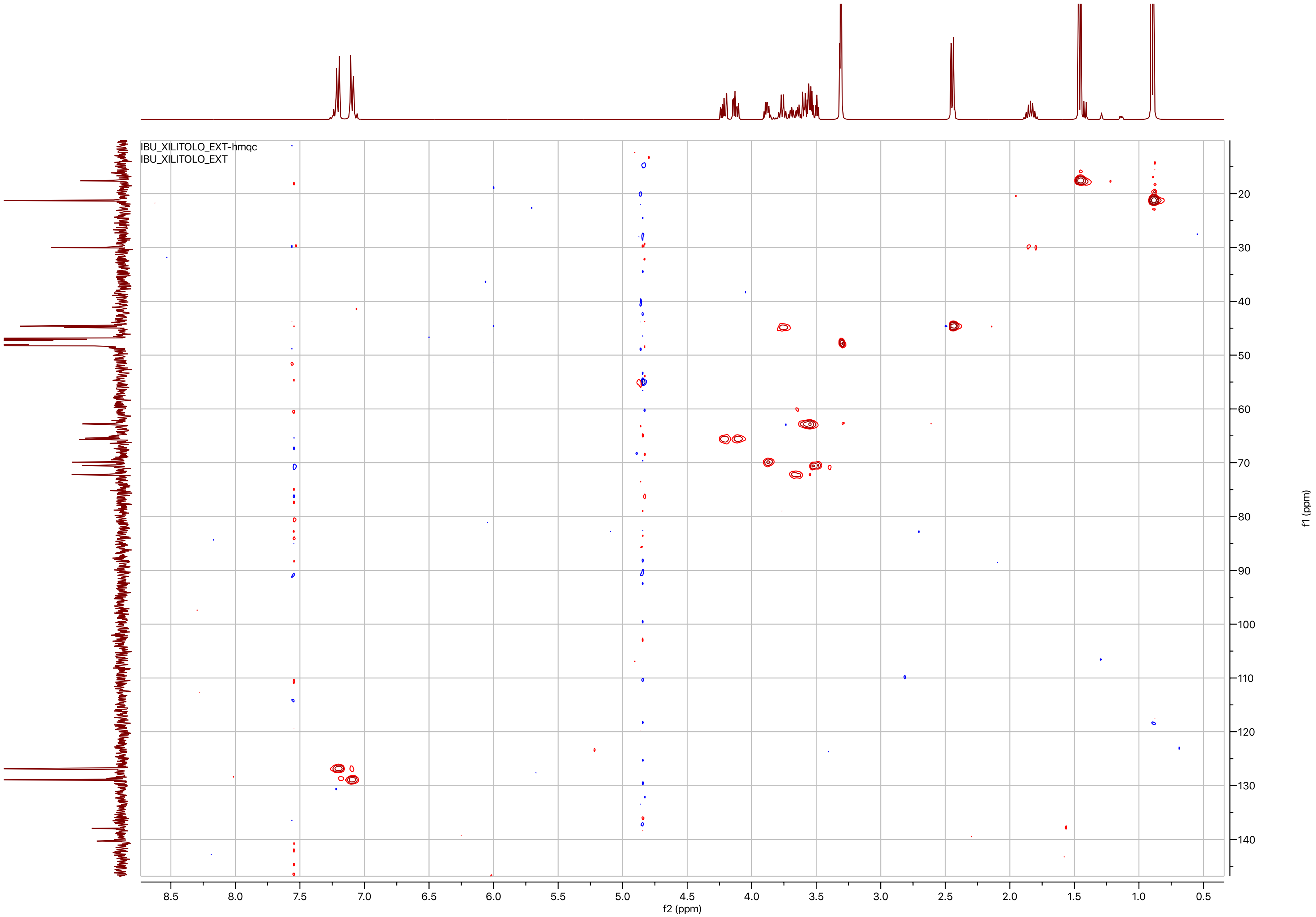

Figure S6: HMQC IBU-xylitol ester

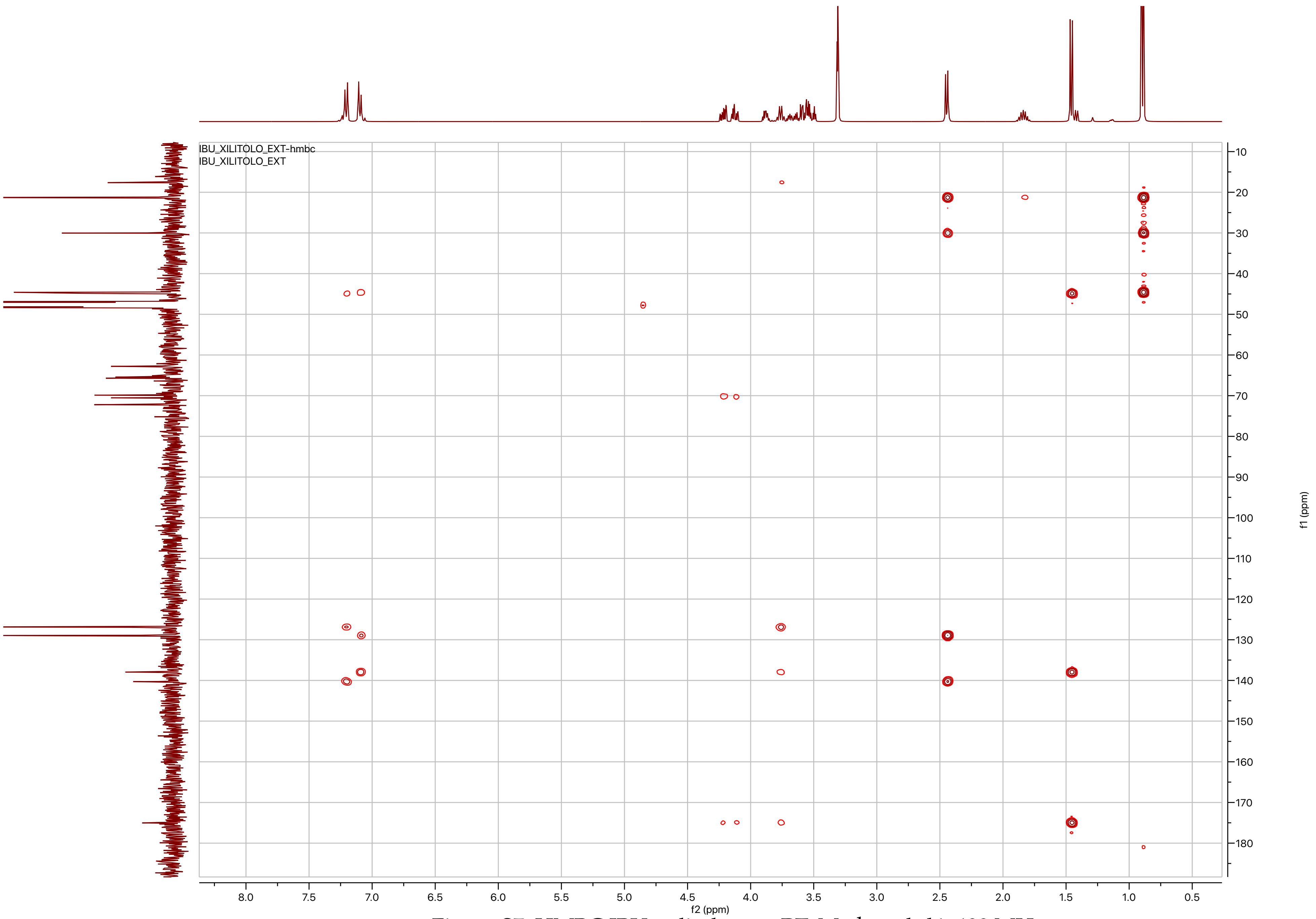

Figure S7: HMBC IBU-xylitol ester; RT, Methanol-d4, 400 MHz.

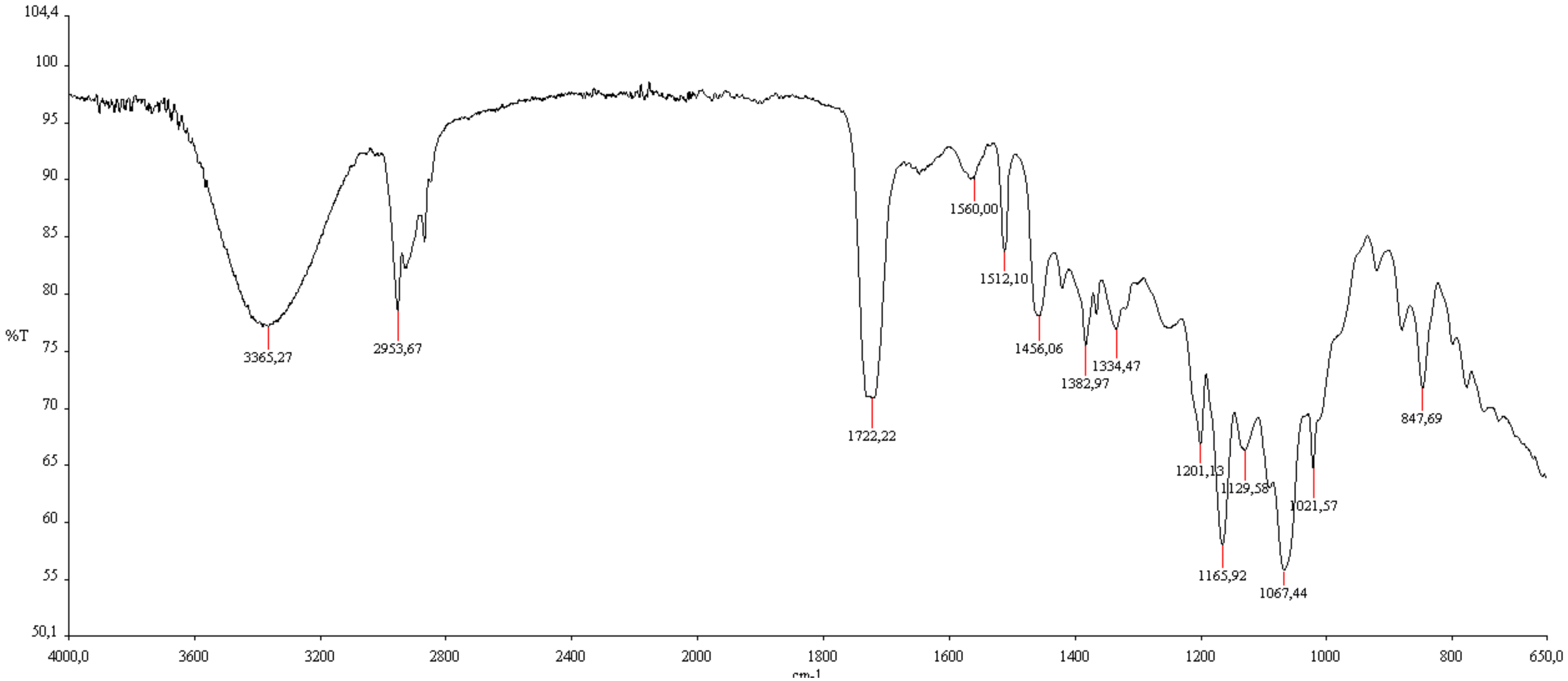

Figure S8: IR spectra of IBU-xylitol ester

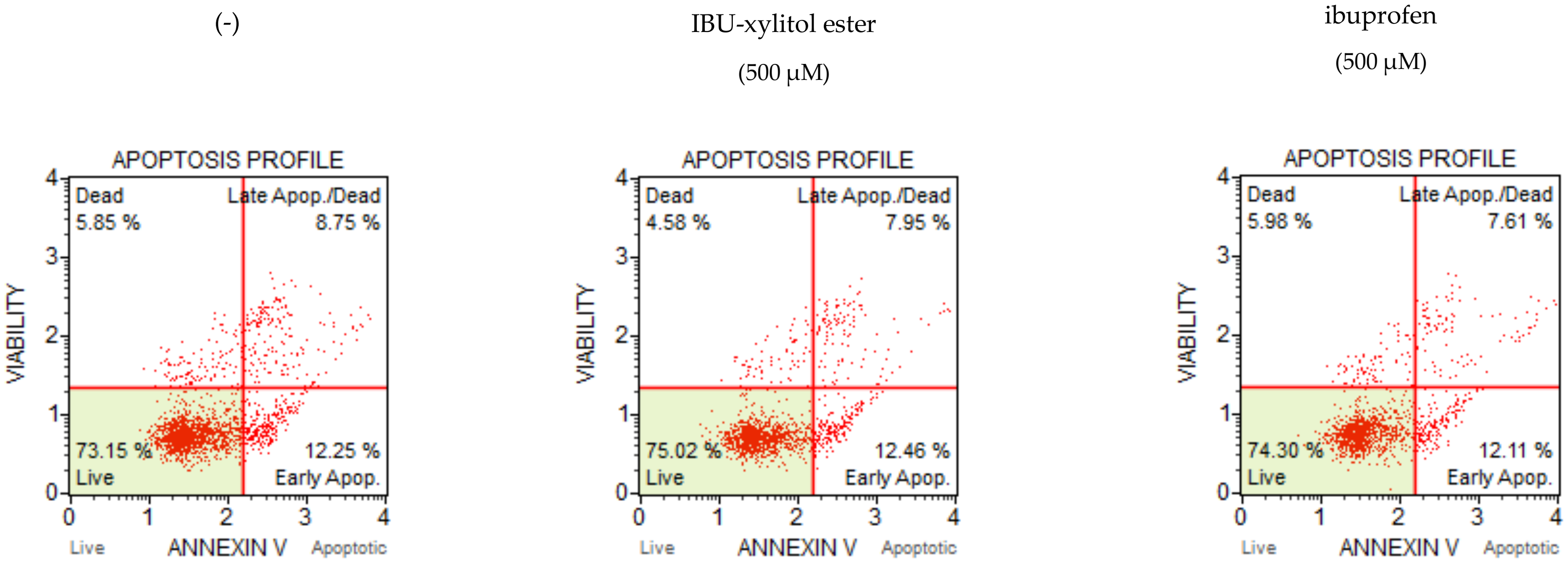

Figure S9: Representative analysis of apoptosis of IB3-1 cells cultured for 24h in the presence of the indicated concentrations of IBU-xylitol ester and ibuprofen. Apoptosis was assessed by the Annexin V assay.
